# The SWI/SNF Chromatin Remodeling Complex Influences Transcription by RNA Polymerase I in *Saccharomyces cerevisiae*

**DOI:** 10.1371/journal.pone.0056793

**Published:** 2013-02-20

**Authors:** Yinfeng Zhang, Susan J. Anderson, Sarah L. French, Martha L. Sikes, Olga V. Viktorovskaya, Jacalyn Huband, Katherine Holcomb, John L. Hartman, Ann L. Beyer, David A. Schneider

**Affiliations:** 1 Department of Biochemistry and Molecular Genetics, University of Alabama at Birmingham, Birmingham, Alabama, United States of America; 2 Department of Microbiology, University of Virginia Health System, Charlottesville, Virginia, United States of America; 3 Department of Genetics, University of Alabama at Birmingham, Birmingham, Alabama, United States of America; 4 The University of Virginia Alliance for Computational Science and Engineering, Charlottesville, Virginia, United States of America; Texas A&M University, United States of America

## Abstract

SWI/SNF is a chromatin remodeling complex that affects transcription initiation and elongation by RNA polymerase II. Here we report that SWI/SNF also plays a role in transcription by RNA polymerase I (Pol I) in *Saccharomyces cerevisiae*. Deletion of the genes encoding the Snf6p or Snf5p subunits of SWI/SNF was lethal in combination with mutations that impair Pol I transcription initiation and elongation. SWI/SNF physically associated with ribosomal DNA (rDNA) within the coding region, with an apparent peak near the 5′ end of the gene. In *snf6*Δ cells there was a ∼2.5-fold reduction in rRNA synthesis rate compared to WT, but there was no change in average polymerase occupancy per gene, the number of rDNA gene repeats, or the percentage of transcriptionally active rDNA genes. However, both ChIP and EM analyses showed a small but reproducible increase in Pol I density in a region near the 5′ end of the gene. Based on these data, we conclude that SWI/SNF plays a positive role in Pol I transcription, potentially by modifying chromatin structure in the rDNA repeats. Our findings demonstrate that SWI/SNF influences the most robust transcription machinery in proliferating cells.

## Introduction

Biosynthesis of ribosomes is proportional to cell growth and proliferation rates. To match the ribosome synthesis rate with the demand for protein synthesis, eukaryotic cells regulate the transcription of rDNA by RNA polymerase I (Pol I). For example, multiple studies have shown that transcription initiation by Pol I is regulated via the initiation factor Rrn3p [1], [2]. Recent work has also identified Pol I transcription elongation as an important target for regulation of rRNA synthesis by UBF and Paf1C [3], [4], [5]. Since rRNA processing is also functionally coupled to Pol I transcription elongation [4], [6], [7], the elongation step in the transcriptional cycle has gained attention.

SWI/SNF is an ATP-dependent chromatin remodeling complex. The SWI/SNF complex was first discovered by screening for genes that control mating type switching (SWI) and sucrose non-fermenting (SNF) phenotypes in yeast [8], [9], [10]. The SWI/SNF complex contains multiple subunits and is conserved among eukaryotes. Though the number and identity of subunits vary between species, eukaryotic cells all contain the DNA-dependent ATPase Swi/Snf2p and the core subunit Snf5p.

Several mechanisms by which SWI/SNF affects the control of gene expression have been proposed. Generally, the SWI/SNF complex mobilizes nucleosomes by utilizing the energy of ATP hydrolysis via the Swi/Snf2p subunit (mammalian orthologs are BRM or BRG1). As a result, the interface between histones and DNA is locally altered, and DNA is rendered accessible to basal transcription machinery [11], [12]. ATP hydrolysis may cause nucleosome sliding, octamer transfer to another DNA molecule, conformational change of nucleosomes, or eviction of histones [11], [13]. A recent biochemical study indicated that SWI/SNF contributes to eviction of one nucleosome of a dinucleosome [14]. SWI/SNF remodels the nucleosome as it translocates along the DNA in one direction [15]. Thus, as the distance between the two nucleosomes shortens, the H2A/H2B dimer of the distal nucleosome is rapidly displaced followed by eviction of the entire histone octamer. Despite a multitude of studies focused on characterizing the mechanism(s) by which SWI/SNF functions, the exact mechanism of action *in vivo* remains to be determined. Considering the internal and external stimuli that cells must confront, it is possible that SWI/SNF employs diverse mechanisms to modify different chromatin states.

Genome-wide studies have shown that SWI/SNF acts both as an activator of transcription and as a repressor in a subset of genes [16], [17], [18]. SWI/SNF-dependent alteration of gene expression is responsive to changing nutrient conditions (rich versus minimal media) and stress conditions (heat shock versus stationary-phase stress). Thus, multiple signaling pathways influence SWI/SNF-mediated control of chromatin structure.

Gene specific studies further demonstrated that SWI/SNF plays a role in activating transcription. Mutation of the genes that encode Swi1p, Swi2/Snf2p, and Swi3p in yeast has been reported to impair transcription induction in a variety of genes including HO, INO1, ADH1, ADH2, SUC2, GAL1, and GAL10 [19], [20], [21]. For example, cell lines that express a catalytically impaired form of Brg1 or hBrm were unable to activate the endogenous stress response gene HSP70 in response to metabolic inhibitors or heavy metals [22]. Snf5p has also been shown to directly bind to the transactivator c-MYC, and mutations in SNF5 or BRG1 abolish the ability of c-MYC to activate transcription [23]. All of these studies show that SWI/SNF is required for targeted activation of gene expression.

In contrast to its roles as a transcriptional activator, SWI/SNF has been suggested to serve as a repressor, though these effects may be indirect. SWI/SNF is involved in repression of SER3 expression [12], [24] and localizes to the promoter of SER3. In the absence of Snf2p, SER3 expression increased more than 50-fold, and overexpression of Snf2p in snf2Δ mutant cells restored normal repression. This role for SWI/SNF may be indirect, since it was later shown that SWI/SNF activates SRG1 (an intergenic transcript adjacent to SER3) which represses SER3 [25]. SWI/SNF is also required for repression of deoxyribonucleotide triphosphate metabolic enzymes during exit from the cell cycle [26]. Moreover, there are reports that link SWI/SNF to repression of the c-FOS proto-oncogene [27]. Mutation in the ATPase domain of BRG1 reduces its capability to repress the transcription of c-FOS. Thus, multiple lines of evidence implicate SWI/SNF in gene repression as well as activation.

Here, we show that SWI/SNF influences transcription by Pol I. Deletion of *SNF5* or *SNF6* was lethal in strains impaired for transcription initiation and elongation by Pol I. ChIP analysis showed that several subunits of SWI/SNF associate with rDNA. Deletion of *SNF6* led to ∼2.5-fold less Pol I transcription than WT. However, there was no change in the density of polymerases per gene or the percentage of actively-transcribed rDNA genes, suggesting a role for SWI/SNF in transcription elongation by Pol I. EM analysis confirmed the unchanged Pol I density through the rDNA coding region and revealed a reproducible peak of Pol I accumulation in the 5′ end of the gene in *snf6*Δ mutant cells relative to WT strains. We conclude from these findings that SWI/SNF plays an important role in Pol I transcription elongation. Based on the known roles for SWI/SNF at other genomic loci, we propose that SWI/SNF may directly affect rDNA chromatin structure.

## Materials and Methods

### Strains and Media used in the Study

Strains used in this study are listed in Table 1. Yeast cells were grown in either synthetic glucose (SD) medium or yeast extract/peptone/dextrose (YEPD) at 30°C with aeration. Recipes for media used were described in detail previously [5]. Where indicated, we have replaced the *S. cerevisiae URA3* gene with the *S. pombe URA4* gene for recombination-mediated gene disruptions and epitope tag fusions. *URA4* encodes a protein with the same function (orotidine-5′-phosphate decarboxylase), however there is limited or no recombination with the endogenous *URA3* locus.

**Table 1:** Strains used in this study.

Strains	Description
NOY388	*MATa ade2-1 ura3-1 trp1-1 leu2-3,112 his3-11,15 can1-100*
NOY396	*MATα ade2-1 ura3-1 trp1-1 leu2-3,112 his3-11,15 can1-100*
NOY2172	*MATa ade2-1 ura3-1 trp1-1 leu2-3,112 his3-11 can1-100 rpa135(D784G)*
DAS50	Same as NOY396, but *rpa49Δ::LEU2*
DAS303	Same as NOY396, but *RRN7*-(MYC)_13_::*TRP1*
DAS477	Same as NOY396, but *RPA135*-(his)_7_-(HA)_3_::*TRP1,MATa*
DAS483	*MATa rpa135(D784G)*::nat*^r^*, *ura3*, *his3*, *leu2*, *can1*Δ::MFa1pr-HIS5sp, *lyp1*Δ
DAS484	*MATα rpa135(D784G)*::nat*^r^*, *ura3*, *his3*, *leu2*, *can1*Δ::MFA1pr-HIS5sp, *lyp1*Δ*/hmr*Δ::*URA3*
DAS648	*ade2-1 ura3-1 trp1-1 leu2-3, 112 his3-11,15 can1-100*
DAS647	Same as DAS648, but *snf6*Δ::*URA4*
DAS649	Same as NOY396, but *SNF6*- MYC_13_::*HIS3*
DAS651	Same as NOY396, but *SNF5*-(his)_7_-(HA)_3_::*URA4*
DAS750	Same as NOY396, but *MAT? SNF2*-(his)_7_-(HA)_3_::*HIS3*
BY4741	*MATa his3Δ1 leu2Δ0 met15Δ0 ura3Δ0*; [57]

### Synthetic Genetic Array (SGA)

SGA was conducted as described previously [28], [29].

### Genetic Interactions

We constructed null mutants of *snf5*Δ and *snf6*Δ in NOY396 using established methods for yeast transformation [30]. Then *snf5*Δ and *snf6*Δ were mated with *rpa135(D784G)* or *rpa49*Δ in YEPD media. Diploid strains were selected on SD-ura-leu for the combination of *rpa49*Δ with *snf5*Δ or *snf6*Δ; combination of *rpa135(D784G)* with *snf5*Δ or *snf6*Δ was selected on SD-ura+neurseothricin. Following sporulation, tetrads were dissected onto YEPD plates using a Zeiss Axioskop 40 Tetrad Microscope. 50–60 tetrads of each genetic background were dissected. The dissection plates were incubated at 30°C >7 days; genotypes of individual segregants were tested by screening for nutritional markers linked to the deletion mutation.

### Chromatin Immunoprecipitation (ChIP)

The procedure was described previously [4] except cells were treated with formaldehyde for 1hr to successfully crosslink SWI/SNF to the rDNA [31]. The detailed ChIP procedure and sequences for primers used in real-time analysis are described in Supplementary Data. ChIP data were quantified from at least 2 10-fold dilutions per sample from duplicate cultures.

### Measurement of rRNA Synthesis

[methyl-^3^H]methionine incorporation assays were performed as described previously [2]. The rationale of this assay is more thoroughly explained in Anderson et al. 2011 [32]. Briefly, we cultured cells in SD-met to exponential phase and pulse-labeled cells for 5 min with 20µCi/ml [methyl-^3^H]methionine followed by a 5 min chase with 500 µg/ml of cold methionine. RNA was extracted, run on a denaturing gel, transferred to a membrane, and detected by autoradiography.

### rDNA Copy Number Determination

rDNA copy number was measured as described previously [4]. Briefly, chromosomes were separated by CHEF, stained with ethidium bromide, transferred to a membrane, hybridized with a ^32^P-dCTP labeled 600 bp rDNA probe [primers used to amplify the probe were provided in [4]], and detected by autoradiography.

### EM Analysis

Miller chromatin spreads and EM analyses were performed as described [33]. Polymerase occupancy measurements are described in El Hage et al. 2010 [34].

### Statistical Analysis

Statistical analyses were performed on all the data from ChIP and EM assays. For EM data, Kolmogorov-Smirnov (K-S) tests were conducted to test the hypothesis that distributions of Pol I on active genes are comparable for *snf6*Δ and WT. The results indicate that the hypothesis can be rejected. For ChIP data, two-way ANOVA tests were performed to test the following hypotheses: i) within a given strain there is little variation across locations, ii) at given locations there is little variation between strains, and iii) there is no interaction between strain and location. The results indicate that all three hypotheses could be rejected. As a follow-up, TukeyHSD tests were performed to test the hypothesis that binding signals are the same between different strains at the same locations. Rejections of this hypothesis were documented, and p-value was provided in this study.

## Results

### Genetic Interactions between snf5Δ or snf6Δ and rrn3-ts, rpa49Δ or rpa135(D784G)

We have previously identified a mutation in the gene encoding the second largest subunit of Pol I that renders the enzyme impaired for transcription elongation (*rpa135(D784G)*; [6]). Thus, mutations in genes that interact with *rpa135(D784G)* would be candidates to influence Pol I transcription elongation. To identify synthetic lethal partners of *rpa135(D784G)*, we performed a synthetic genetic array (SGA) screen [29]. To increase the reliability of our screen, we constructed Mat a and Mat α strains carrying the *rpa135(D784G)* mutation (DAS483 and DAS484) and screened using both mutant libraries for synthetic lethal interactions. We identified and confirmed 17 synthetic lethal interactions. Of particular interest, we found deletion of *SNF6* to be synthetic lethal with *rpa135(D784G)*.

**Figure 1:**
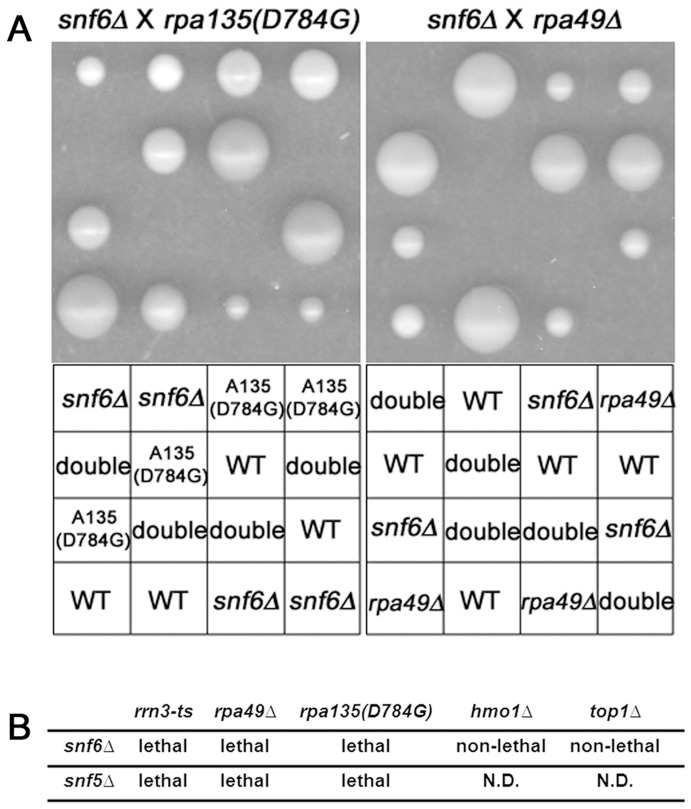
The SWI/SNF complex interacts genetically with Pol I. (A) Deletion mutants of *SNF6* (DAS647) were mated with *rpa135(D784G)* (NOY2172) or *rpa49*Δ (DAS50). Diploids were selected, sporulated, and resulting tetrads were dissected. Images of haploid colonies were made after 2 days incubation at 30°C. Segregant genotype is labeled below each image. (B) Genetic interactions between indicated strains are listed. Non-lethal interactions were confirmed to have predicted growth rates, taking into account the defects in growth present in parental haploid strains. “N.D.” indicates genetic combinations that were not analyzed.

To confirm this synthetic lethal interaction, we deleted *SNF6* in a different strain background [W303-1A (NOY 388)] and tested for interaction with the *rpa135* mutation using tetrad dissection. Of 48 total double mutant segregants [*snf6*Δ *rpa135(D784G)*], none germinated even after 7 days of incubation at 30°C (Figure 1A), confirming SWI/SNF function is required when Pol I transcription elongation is impaired.

To determine if this genetic interaction was specific to the *rpa135(D784G)* allele or a more general characteristic of impaired Pol I transcription elongation, we mated a *snf6*Δ strain to a strain carrying a deletion of *RPA49*. The A49 subunit of Pol I (also referred to as Rpa49p) is not essential for growth; however, A49 forms a heterodimer with the A34.5 subunit (also referred to as Rpa34p) that acts as an intrinsic transcription elongation factor [35]. Therefore, genetic interactions between *snf6*Δ and *rpa49*Δ would further support the model that SWI/SNF plays a role in the elongation step of Pol I transcription.

Tetrad analysis revealed that the combination of *snf6*Δ with *rpa49*Δ was also lethal. None of 55 haploid double mutants grew even after long term incubation (Figure 1A). Since the A49 subunit and the *rpa135(D784G)* allele influence transcription elongation by Pol I, these interactions could support a role for *SNF6* in the elongation step of Pol I transcription. However, we also performed tetrad analysis of a cross between *snf6*Δ and *rrn3-ts*. Rrn3p is a well characterized transcription initiation factor for Pol I. We found that *snf6*Δ is lethal with *rrn3-ts* (Figure 1B). Thus, mutations that substantially impair transcription initiation or elongation by Pol I are synthetic lethal with *snf6*Δ. These data suggest a direct or indirect role for SWI/SNF in transcription by Pol I, but cannot implicate any single step in the transcription cycle. We note that not all genes whose products influence rRNA metabolism interacted with the *snf6* deletion. Neither *hmo1*Δ nor *top1*Δ showed significant interactions with *snf6*Δ.

To further investigate whether the genetic interaction with Pol I is unique to *SNF6* or a general property of the SWI/SNF complex, we included Snf5p [a core subunit of SWI/SNF] in our analysis. Similarly, we found that *snf5*Δ was lethal in combination with *rrn3-ts*, *rpa135(D784G)* or *rpa49*Δ (Figure 1B). These genetic interactions demonstrate that the SWI/SNF complex plays an important role in Pol I transcription, influencing transcription initiation and/or elongation.

### Subunits of SWI/SNF Associate with rDNA

**Figure 2:**
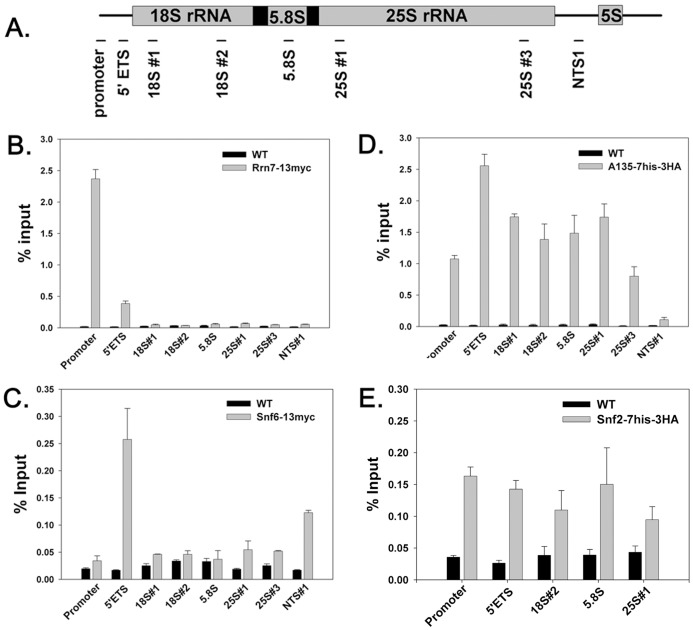
SWI/SNF associates with rDNA. Yeast cells were cultured, crosslinked with formaldehyde, lysed, sonicated and immunoprecipitated with anti-Myc 9E10 or anti-HA 12CA5 antibody. (A) Positions of 8 pairs of primers used in real-time PCR analyses are indicated by horizontal bars under the schematic of an rDNA repeat. (B) Positive control [Rrn7-(MYC)_13_ (DAS303)] and negative control [WT (NOY396)] ChIP results. Y-axis represents the amount of rDNA immunoprecipitated relative to total rDNA in input samples. (C) Snf6p (DAS649) ChIP signal compared to negative control. (D) A135-(his)_7_-(HA)_3_ (DAS477) associates with the promoter and the entire coding region of rDNA. (E) Snf2p (DAS750) associates with the coding region of the rDNA. Data were quantified from at least 2 10-fold dilutions per sample from duplicate cultures. Error = ±1standard deviation.

To investigate the direct relevance of SWI/SNF in Pol I transcription, chromatin immunoprecipitation (ChIP) assays followed by real-time PCR were designed to detect association of Snf2p, Snf5p and Snf6p with rDNA using 5–8 pairs of primers located throughout the rDNA repeats (Figure 2A and File S1). We tagged Snf2 and Snf5p with a 7his-3-hemagglutinin (HA) epitope on the C-terminus and tagged Snf6p with a 13-Myc epitope on the C-terminus in WT background (Table 1). Neither tag affected growth rate. Rpa135-(his)_7_-(HA)_3_ and Rrn7-(Myc)_13_ were used as positive controls. The untagged parental strain was used as a negative control. Snf6-(Myc)_13_ ChIP to *GAL1* served as a positive control for interaction with a Pol II transcribed gene (Figure S1 in File S1).

*RRN7* encodes a subunit of core factor, an essential transcription initiation factor for Pol I. As expected, Rrn7p specifically bound rDNA at the promoter (Figure 2B). Residual signal in the 5′ external transcribed spacer (ETS) region is often observed for transcription initiation factors, due to the proximity of this region to the promoter. Statistical analysis, using TukeyHSD tests, confirms that the binding signals at the promoter and 5′ ETS are significantly higher than in the untagged control (WT) (with p-value <0.00005). In contrast, Rpa135-(his)_7_-(HA)_3_ bound to the transcribed region of the rDNA with statistical significance (p-value <0.00005) but not to the non-transcribed spacer (NTS) regions (Figure 2D) [4]. As expected, Snf6p binds to Pol II transcribed *GAL1* (Figure S1 in File S1). As seen in Figures 2C, Snf6p specifically bind rDNA in the 5′ ETS. Although the signal for Snf6p in the 5′ ETS is lower than for positive controls, it is at least 3-fold above the background signal (untagged WT). Statistical analysis confirms that the association of Snf6p with 5′ ETS is significantly higher than WT (TukeyHSD Test with p-value <0.00005). Unlike Snf6p, Snf2p detectably associates with the entire rDNA from the promoter through the rDNA coding regions (Figure 2E). We also tagged Snf5 and observed a small peak in the 5′ ETS, however, though qualitatively consistent with our Snf6 data (Figure 2C) this association was not statistically significant (Figure S2 in File S1). In summary, SWI/SNF associates with the rDNA and is poised to affect Pol I transcription.

Snf6p and Snf5p also associate with the NTS regions of the rDNA. Several transcription factors have been reported to bind the NTS regions including Spt4/Spt5 [7], Chd1p and Isw1p [36], and Paf1C [4]. These positive ChIP signals are thought to be due to the association of transcription factors with Pol II transcription units within the spacers. Although the functional consequence of SWI/SNF association with the NTS region is not yet clear, its association with the rRNA coding region is consistent with a model whereby SWI/SNF could directly influence Pol I transcription.

We note that the ChIP signal for Snf2p, Snf5p and Snf6p is substantially lower than is observed for proteins that associate with Pol II-transcribed genes. This observation is consistent with many previous observations from our lab and others. One potential explanation for this observation is that the dense organization or assembly of pre-ribosomes on the nascent rRNA may influence IP efficiency under standard ChIP conditions. Furthermore, since epitope availability is certainly different between subunits of the complex and can vary in response to nearby protein density, the difference in ChIP signal between Snf6 and Snf2 does not necessarily indicate any biologically relevant difference in the complex composition or occupancy. From all of these data (Figures 2 and S2) we simply conclude that SWI/SNF is in close proximity to the rDNA, rendering it capable of influencing Pol I.

### Deletion of SNF6 Decreases Pol I Transcription Rate *in vivo*

**Figure 3:**
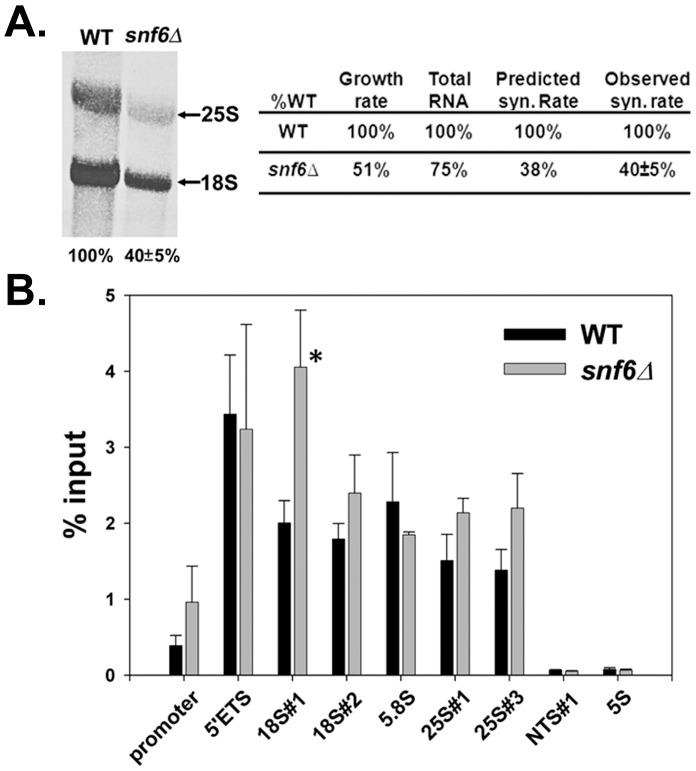
rRNA synthesis rate of *snf6*Δ is only 40% of WT despite similar Pol I occupancy. (A) Yeast cells were cultured in SD-met media to an A_600_ ∼ 0.3. Then [methy-^3^H]methionine was incorporated into cells for 5 min followed by a 5 min chase with excess (500 µg/ml) cold methionine. RNA was isolated, run on an agarose gel, transferred to nitrocellulose membrane, and detected by autoradiography. Pol I transcription in *snf6*Δ was normalized to WT. Data were analyzed from 2 independent [methy-^3^H]methionine incorporation assays. Only one dataset is shown here. (B) Pol I occupancy of rDNA in *snf6*Δ resembles that of WT by ChIP except for a 2-fold increase of Pol I occupancy in *snf6*Δ at the 5′ end of the 18S gene (asterisk). An anti-A190 polyclonal antibody was used to immunoprecipitate Pol I complexes. The 8 primer pairs described for Figure 2 were used in real-time PCR to check the association of Pol I complex with rDNA. A ninth primer pair measured Pol I association with 5S rDNA. Data were quantified from at least 2 10-fold dilutions per sample from duplicate cultures. Error = ±1standard deviation.

To assess whether *snf6*Δ decreases Pol I transcription rate, we pulse labeled yeast cells with [methyl-^3^H]methionine [2]. Because rRNA in yeast is co-transcriptionally methylated and the pool of methionine within living cells is low, the [methyl-^3^H]methionine incorporation assay is a reliable method to quantify rRNA synthesis rates [37]. As shown in Figure 3A, the rRNA synthesis rate in the *snf6*Δ mutant was 40% of the WT rate.

In our experience, the [methyl-^3^H]methionine incorporation assay is generally not influenced by alteration in isotope uptake or fluctuations in the cellular methionine pool between strains. Isotopic labeling with uridine, for example, is prone to these indirect effects. However, it is formally possible that *snf6*Δ-dependent changes in methionine pools or activity of methyltransferases could be incorrectly interpreted as defects in rRNA synthesis. To control for this possibility, we calculated the predicted rRNA synthesis rate by considering the cellular growth rate and total RNA accumulation of WT and *snf6*Δ cells in liquid culture. This prediction assumes minimal or no rRNA degradation. The *snf6*Δ mutant grew only 50% as fast as WT. Furthermore, the total RNA level in *snf6*Δ was only about 75% of WT (data not shown). Based on these values, one would predict an rRNA synthesis rate of 37.5% of WT in *snf6*Δ cells [0.5×0.75 = 0.375; Figure 3A; see Supplementary data for additional explanation of this calculation]. This predicted rate is very close to the rate determined directly by [methyl-^3^H]methionine. Thus, our measurement of rRNA synthesis rate is reliable and accurate. These data support the model that SWI/SNF affects one or more rate limiting steps in Pol I transcription.

As an additional control we measured total RNA synthesis in WT and *snf6*Δ cells using ^3^H-uridine labeling. Uridine incorporation detected a large defect in transcription in the *snf6*Δ cells compared to WT (∼25-fold; Figure S3 in File S1). Such a severe defect in rRNA synthesis would not support growth, especially not at the rates observed (Figure 3A). Since fluctuations in the cellular uridine pool can dramatically affect this experimental approach, the magnitude of this effect is not reliable; however, these data provide additional qualitative support that mutation of *SNF6* affects rRNA synthesis.

Reduced accumulation of rRNA might also result from an enhanced decay rate rather than a reduction in transcription rate. To test whether accelerated degradation of RNA in *snf6*Δ mutant cells contributed to the observed decrease in labeled 18S and 25S rRNA, we constructed a *snf6*Δ *rrp6*Δ double mutant and reexamined rRNA synthesis using [methyl-^3^H]methionine. Rrp6p is a subunit of the nuclear exosome and is required for efficient degradation of excess and aberrant rRNAs and mRNAs [38], [39], [40]. We have shown previously that mutation of *RRP6* leads to accumulation of unstable rRNA precursors and degradation products [6]. We postulated that if increased exosome-dependent rRNA degradation rate was accounting for decreased rRNA, deletion of *RRP6* would restore rRNA levels in the mutant cells. However, the amount of rRNA in *snf6*Δ *rrp6*Δ double mutant was not higher than in *snf6*Δ alone (Figure S4 in File S1), thus we concluded that rRNA degradation did not contribute significantly to the decrease in the rRNA synthesis observed in the *snf6Δ* strain. These data argue against involvement of exosome-dependent RNA degradation in influencing rRNA levels in *snf6*Δ cells. Rather, we conclude that SWI/SNF promotes Pol I transcription.

### Deletion of SNF6 does not Affect Pol I Occupancy of rDNA

We postulated that if Snf6p primarily affects transcription initiation by Pol I, we would observe a dramatic decrease in Pol I occupancy of rDNA in *snf6*Δ mutant cells relative to WT. To assess the effect of Snf6p on Pol I transcription initiation, we measured Pol I occupancy of the rDNA using ChIP. Our data showed that Pol I occupancy of the rDNA repeat did not decrease in *snf6*Δ compared to WT (Figure 3B). Furthermore, there was a significant elevation of Pol I signal at one location, position 18S#1 in the *snf6*Δ mutant compared to WT (TukeyHSD test with p-value <0.0005) (Figure 3B, asterisk). This observation suggests that Pol I may transiently stall or arrest in the 5′ end of rDNA in *snf6*Δ mutant cells. Together, these data demonstrate that impaired transcription initiation alone cannot account for the defect in rRNA synthesis observed in the *snf6*Δ strain, since impaired initiation alone would result in lower Pol I occupancy of rDNA (see discussion below).

### Deletion of SNF6 does not Affect rDNA Copy Number

Eukaryotic rDNA exits as tandem repeats that can rapidly and efficiently recombine to alter the number of repeats present in any given cell [41]. Thus, one must control for mutation-induced alteration in the rDNA copy number. We measured relative rDNA copy numbers in *snf6*Δ mutant cells compared to WT by contour-clamped homogenous field electrophoresis (CHEF) followed by Southern blot hybridization as described previously [4] (Figure S5 in File S1). We included control strains with known alterations of rDNA copy number. There was no reduction in the rDNA copy number in *snf6*Δ mutant compared to WT (Figure S5 in File S1). Thus, decreased Pol I transcription in the *snf6*Δ mutant is not due to alteration of the rDNA copy number.

Mutation of *SNF6* impairs rRNA synthesis; however, Pol I occupancy of rDNA is unchanged, RNA degradation by the exosome is normal, and there is no alteration in the rDNA copy number. The simplest explanation for these data is that SWI/SNF affects transcription elongation by Pol I.

### Mutation of SNF6 does not Alter the Number of Polymerases Engaged in Transcription

To further test our model that Snf6p acts in Pol I transcription elongation, we used electron microscopy of Miller chromatin spreads (EM analysis) to visualize Pol I transcription *in vivo*. EM analysis permits quantification of Pol I density per gene, percentage of actively transcribed genes, and Pol I positioning over the gene.

**Figure 4:**
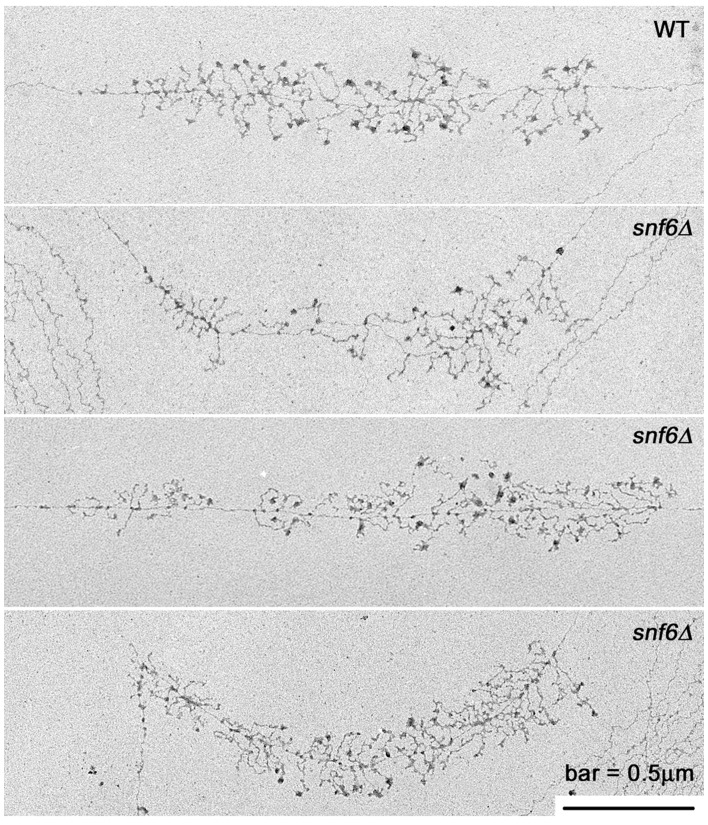
Miller chromatin spreads of WT and *snf6*Δ suggest similar Pol I occupancy of rRNA genes. Direction of transcription is from left to right. rRNA transcripts per rDNA gene were quantified for these representative and many additional genes. For the genes shown, the number of rRNA transcripts was 51 for WT (DAS648), and was 44, 39, and 60 for the three *snf6*Δ (DAS647) genes (top to bottom).

**Figure 5:**
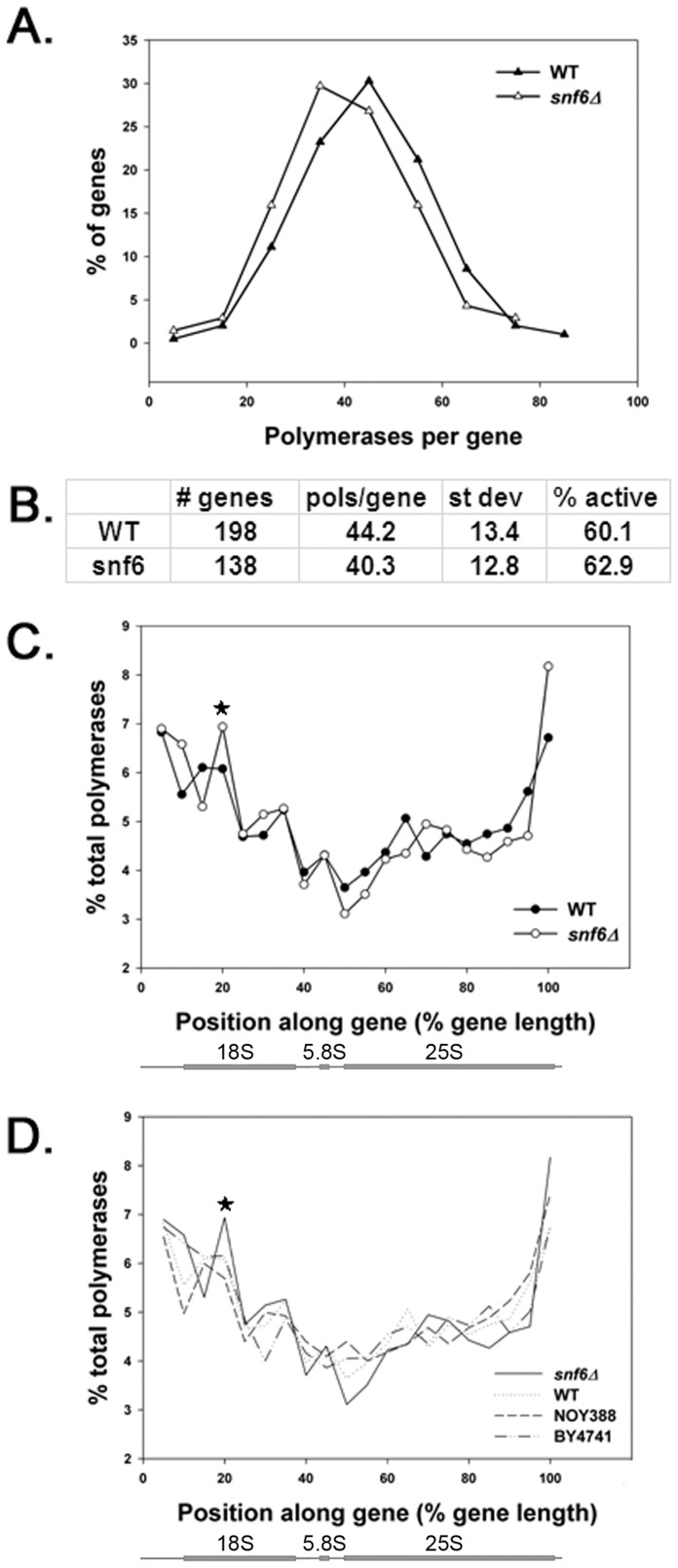
A small accumulation of Pol I complexes in the 5′ end of rDNA in *snf6*Δ. (A) Distribution frequency for the number of polymerases per gene was revealed by EM analysis of Miller chromatin spreads in *snf6*Δ (DAS647) and WT (DAS648). (B) More than 100 rDNA genes from Miller chromatin spreads were analyzed in WT and *snf6*Δ. Pol I density and percentage of actively transcribed genes in *snf6*Δ and WT are similar. (C) Polymerase occupancy as a function of position within the transcribed region of rDNA in *snf6*Δ and WT cells. A small peak of Pol I occupancy in the 5′ end of rDNA in *snf6*Δ is indicated by an asterisk. All mappable genes in the dataset were analyzed, corresponding to >60 genes per strain and >2500 polymerases per strain. Schematic below the X-axis represents the Pol I transcribed region of the rDNA. (D) Same as C but an additional two WT control strains (NOY388 and BY4741) are plotted. BY4741 data are from El Hage et al. 2010 [34].

Representative rDNA repeats are shown in Figure 4 for WT and *snf6*Δ cells. We analyzed >60 genes per strain (>2500 polymerases per strain) to quantify Pol I occupancy of the rDNA. We concluded from quantification of the EM data (Figure 5A and 5B), that Pol I occupancy of the rDNA was not increased or decreased significantly in the *snf6Δ* strain compared to WT, consistent with our ChIP data (Figure 3B). Since impaired transcription elongation without any decrease in initiation rate would result in increased Pol I occupancy, these data indicate that both Pol I initiation and elongation rate are impaired, but that initiation rate was not inhibited more severely than elongation in the mutant cells.

Not all rDNA repeats are actively transcribed at any one moment. Consequently, the percentage of actively transcribed rDNA repeats can affect overall rRNA synthesis rate. However, because EM data showed that ∼60% of rDNA repeats are transcribed in both *snf6*Δ and WT cells (Figure 5B), mutation of SWI/SNF does not influence the “on-off” status of rDNA repeats.

There is no method for direct measurement of transcription elongation by Pol I *in vivo,* however, we can estimate the relative elongation rates in different strains based on the data presented. We calculate the number of engaged Pol I complexes using the following equation: engaged Pol I complexes = (pols/gene) * (rDNA copy number) * (percentage of actively transcribed rDNA). We found each parameter in the equation to be approximately equal in WT versus *snf6*Δ, therefore we conclude that a similar number of Pol I complexes is engaged in transcription in WT and *snf6Δ* cells. Since the synthesis rate of rRNA in the *snf6Δ* strain is only 40% of the WT level (based on [methyl-^3^H]methionine incorporation data) we conclude that the Pol I initiation and elongation rate in *snf6*Δ must be ∼40% of WT.

### Mutation of SNF6 Changes the Distribution of Pol I on Active Genes

ChIP data suggested that there may be a site in the 5′ end of rDNA where Pol I accumulates in *snf6*Δ mutant cells (Fig. 3B). To further test for such a pause or arrest site, we reexamined the EM data and mapped Pol I distribution along rDNA for the genes in our dataset (>2500 pols mapped per strain). We observed a reproducible increase in polymerase density ∼15–20% into the gene compared to the control strain (Figure 5C). Although this increase in polymerase density is relatively small, it is often noticeable by visual inspection of individual genes from *snf6*Δ cells (Fig. 4), and it is consistent with the Pol I peak observed by ChIP at position 18S#1 (Figure 3B). Both of these peaks are near the position of the most robust ChIP signal for Snf6p and Snf5p (Figures 2 and S2). To further test whether this peak in polymerase occupancy is significant, we mapped the Pol I distribution in two additional control strains of different genetic backgrounds. Both lacked a peak in Pol I density at this position. Kolmogorov-Smirnov tests indicated that the distribution of Pol I in the *snf6*Δ strain is statistically different from all the control strains (p-value <0.05) (Figure 5D). Statistical analysis identified a bimodal distribution of Po I in all four strains with one mode in the 15–20% range, and another should appear in the 68–75% range. However, the larger values of the standard deviations when computing the second mode prevent the exact location of that peak. Though it seems Pol I occupancy in ITS and 25S regions is reduced in *snf6*Δ relative to WT (Figure 5C and 5D), our statistical analysis does not support this. Pol I occupancy measured by ChIP also did not identify such reduction (Figure 3). However, identification of modes in the data confirms that the peak in the 5′ end of rDNA is significant. Taken together, the simplest explanation of these data is that SWI/SNF normally functions to promote transcription elongation by Pol I. In the absence of efficient SWI/SNF function, a transient kinetic barrier to elongation exists in the 5′ end of rDNA. The potential significance of this barrier is discussed below.

### Deletion of SNF6 Modestly Affects rRNA Processing

Transcription elongation by Pol I is functionally coupled to efficient rRNA processing. Strains carrying mutations in *RPA135*, *SPT4/5* or *PAF1* had reduced rRNA synthesis rates and significant defects in rRNA processing [4], [6]. Since factors that influence transcription elongation also have the ability to affect rRNA processing, we investigated whether SWI/SNF can influence the efficiency of rRNA processing. No obvious differences were seen in co-transcriptional RNA processing events, such as SSU processome formation, as visualized in Miller chromatin spreads (examples in Figure 4). For more quantitative data, we analyzed pre-rRNA and mature rRNA transcripts by Northern blot (Figure S6 in File S1). We prepared total RNA from *snf6*Δ and WT cells. The ratio of pre-rRNA species compared to mature rRNA was quantified in WT and *snf6*Δ samples (Figure S6 in File S1).

From these studies, we conclude that disruption of *SNF6* had no effect on the processing of the 18S rRNA. We did not detect any alteration of 23S or 20S pre-rRNA in *snf6*Δ mutant cells. However, there was a ∼60% increase of 27S pre-rRNA in the mutant compared to WT. We also observed a small increase in the abundance of the unprocessed 35S rRNA species in the mutant compared to WT (Figure S6A in File S1). Thus, there appear to be modest defects in rRNA processing in the absence of Snf6p. Since SWI/SNF affects Pol I transcription elongation, these data are consistent with the model that Pol I elongation and rRNA processing are functionally coupled [6].

We note that a large defect in pre-rRNA processing could lead to a decrease in rRNA accumulation in pulse-chase studies. However, we did not observe accumulation of pre-rRNA when the nuclear exosome was mutated (Figure S4 in File S1) or in pulse-chase isotopic labeling experiments (Figure S7 in File S1). Thus, pre-rRNA processing is not sufficiently defective in *snf6*Δ cells to account for the observed defects in rRNA synthesis.

## Discussion

The work presented above describes a chromatin remodeling complex affecting Pol I transcription elongation. The data show that SWI/SNF interacts genetically with Pol I and physically with the rDNA. Deletion of *SNF6* significantly reduces the Pol I transcription rate independently of changes in rDNA copy number, exosome-dependent rRNA degradation or percentage of active repeats. Although we cannot exclude indirect effects of the *snf6Δ* mutation on Pol I activity, the simplest interpretation of all of these data is that SWI/SNF plays a direct, positive role in transcription by Pol I. We speculate that SWI/SNF may promote clearance of a chromatin-mediated pause in the 5′ end of the coding region of rDNA and facilitate recruitment of additional positively acting factors to the elongation complex.

Considering the robust roles for SWI/SNF in Pol II transcription, it is also possible that SWI/SNF contributes to transcription by Pol I indirectly through altering Pol II-dependent gene expression. However, the ChIP data presented here suggest that SWI/SNF is properly positioned to affect Pol I transcription directly. Thus, the simplest model we can propose is that SWI/SNF directly affects transcription by Pol I.

### Potential Mechanisms by which SWI/SNF may Influence Pol I Transcription

SWI/SNF is directly involved in the mechanisms of transcription initiation and elongation by Pol II [42], [43], [44], [45]. Here, we show that this chromatin remodeler may also directly affect transcription initiation and elongation by Pol I. ChIP analysis identified a peak in association of both Snf5 and Snf6 with the 5′ end of rDNA. EM and Pol I occupancy data revealed a small peak of Pol I accumulation in the 5′ end of the rDNA in the absence of Snf6p, but no enhanced occupancy in other positions. These data suggest that SWI/SNF normally functions to clear one or more pause sites in the 5′ end of the gene.

We do not know the nature of this putative pause. In principle, it could result from DNA sequence specific effects on transcribing polymerases or via a nucleoprotein mediated barrier. Pol I may transiently pause or slow down upon confronting this roadblock. Our data also suggest that there is a general decrease in the elongation rate of Pol I complexes throughout the gene in snf6Δ cells. The Proudfoot lab has shown previously that nucleosomes occupy the entire rDNA, but they lack the regular chromatin structure as in Pol II-transcribed genes [36]. Thus, one explanation for our results is that SWI/SNF participates in clearance of unphased nucleosomes throughout the rDNA repeat. A different study concluded that actively transcribed rDNA repeats lack nucleosomes, but associate with high-mobility group protein Hmo1 [46]. If the latter model is accurate, then SWI/SNF would have a histone-independent role at the rDNA. We have performed micrococcal nuclease mapping experiments in an attempt to identify alterations in the rDNA chromatin in the *snf6Δ* strain relative to WT. Although minor differences were detected, none of these differences was sufficiently clear to differentiate these potential models (data not shown). It is likely that the repeated nature of the rDNA and the maintenance of active and inactive rDNA repeats limit the utility of existing methods in detection of small changes in active rDNA chromatin. Further characterization of the roles that SWI/SNF and other factors play in rRNA synthesis should lead to a better understanding of rDNA chromatin structure.

It has been suggested that SWI/SNF plays a role in facilitating promoter escape by Pol II [promoter escape is a stage in transcription during which polymerases leave the promoter region and engage in productive transcription [47]]. During the first 30 min of induction of Gcn4 expression, Pol II occupancy is not decreased at the ARG1 promoter in snf2Δ mutant cells, but it is decreased at the 3′ end of the ORF, suggesting a role for SWI/SNF in promoter escape. We found no decrease in Pol I occupancy of the promoter or rDNA coding region in snf6Δ compared to WT, indicating that promoter escape by Pol I is not impaired by mutation of SWI/SNF.

In the snf6Δ strain, polymerase density per gene was very similar to WT. For the polymerase occupancy to remain unchanged in the context of a 2.5-fold decrease in synthesis rate, the initiation rate and the elongation rate must have been reduced by approximately the same magnitude in the two strains. The reduction in initiation could be a consequence of defects in transcription elongation (i.e. slower Pol I clearance from the 5′ end of the gene slows loading of the next polymerase). Alternatively, mutation of SNF6 may have independent effects on transcription elongation and initiation (perhaps by altering expression of one or more Pol I transcription initiation factors). The latter model requires alteration of multiple steps in transcription by the same magnitude.

We have previously observed Pol I accumulation in unique regions of the rDNA when elongation is impaired [4]. EM analysis revealed frequent large gaps between transcribing Pol I complexes in a strain carrying a deletion of the gene that encodes the Ctr9p subunit of Paf1C (Polymerase-associated factor complex 1) [4]. Often, upstream of the gap we observed increased polymerase density. The data presented here are distinct from previous observations in the *ctr9Δ* strain. First, the positions of most frequent pausing observed in the *ctr9Δ* cells do not correlate with the position of enhanced Pol I accumulation observed in *snf6Δ* cells ([4] and Figure 5). Second, we did not observe a dramatic increase in gaps between transcribing polymerases in the *snf6Δ* strain. Thus, mutation of genes that encode subunits of Paf1C or the SWI/SNF complex affects Pol I distribution on the rDNA in different ways. Furthermore, these mutations do not interact genetically with one another (data not shown). These observations suggest that there are multiple, discrete mechanisms by which Pol I can be influenced during transcription elongation.

A recent real-time analysis of transcription kinetics at the single cell level reported that transcription elongation is the deterministic step for Pol II transcription whereas initiation is stochastic [48]. Many data demonstrate that transcription initiation by Pol I is regulated; however, several recent studies have implicated transcription elongation factors in the control of Pol I activity [49], [50]. The relative contributions of factors that control transcription initiation, elongation and rRNA decay to the overall regulation of ribosome synthesis remain unclear.

Although our data indicate that SWI/SNF is positioned to directly affect Pol I transcription, we cannot exclude indirect models in which depletion of Snf6p impedes Pol II-dependent expression of genes, which in turn impair Pol I transcription.

### Efficient rRNA Processing Requires Efficient Pol I Transcription Elongation

Coupling of Pol I transcription elongation to rRNA processing has been reported multiple times [3], [4], [6], [7]. The data presented here suggest that mutation of *SNF6* results in a decrease in the amount of rRNA produced per transcribing RNA polymerase I per unit time. Thus, Pol I transcription elongation rate is apparently reduced. Consistent with the model that elongation is coupled to rRNA processing, we observed modest defects on rRNA processing in the *snf6*Δ strain. Thus, these data support the functional linkage between rRNA transcription and rRNA processing.

### Role of SWI/SNF in rDNA Silencing

Previous studies have shown that Pol II-dependent transcription of reporter genes is epigenetically silenced when the reporters are located in the telomeres, the mating-type locus and the ribosomal DNA [51], [52], [53], [54]. SWI/SNF was shown previously to influence silencing of RNA polymerase II-dependent transcription of reporter genes located in the rDNA [55]. The *SUC2* gene normally depends on SWI/SNF for its transcription; however, when it is artificially located in the 25S rDNA coding region, its transcription is instead repressed by SWI/SNF. We suggest that this repression by SWI/SNF is indirect, due to activation of Pol I transcription (leading to occlusion of the Pol II reporter gene). Two additional lines of evidence support this interpretation.

First, the mechanism of SWI/SNF- mediated repression of the reporter is independent of the NAD-dependent histone deacetylase Sir2p or the histone methyltransferase Set1p (36). The *snf2*Δ mutation (Snf2p is the ATPase of SWI/SNF complex) causes several fold higher induction of *SUC2* mRNA than *sir2*Δ and *set1*Δ in this construct. Double mutants of *snf2*Δ*sir2*Δ and *snf2*Δ*set1*Δ induce similar levels of *SUC2* mRNA as in *snf2*Δ single mutant. Moreover, loss of *SNF2* does not affect the levels of histone H3 K4 methylation or the association of Sir2p and Set1p with the rDNA repeat. Thus, SWI/SNF regulates rDNA silencing independently of Sir2p and Set1p.

We did not observe any alteration in the ratio of active versus inactive rDNA repeats in the *snf6Δ* strain compared to WT. The Nomura lab showed previously that rDNA chromatin structure favored by Pol I represses Pol II reporter gene inserted in the rDNA array. They observed that impairment of Pol I transcription leads to induction of Pol II reporter genes located in the rDNA array [56]. All of these data suggest that WT SWI/SNF functions to enhance Pol I transcription elongation, which in turn leads to inhibition of Pol II reporters located in the rDNA coding region.

In summary, our studies demonstrate that SWI/SNF plays important roles in Pol I transcription. Future studies are needed to further define the mechanisms by which SWI/SNF affects Pol I and Pol II functions differentially.

## Supporting Information

File S1 **Seven supplemental figures are provided.** These data include additional controls for ChIP analyses, ChIP analysis of Snf5 association with the rDNA, analyses of rRNA synthesis rates in additional strains, controls for rDNA copy number and characterization of rRNA processing in WT and *snf6Δ* strains. Furthermore, thorough descriptions of methods employed for ChIP and rRNA synthesis measurements are included.
(DOCX)
