# Supplementary material for: The SWI/SNF Chromatin Remodeling Complex Influences Transcription by RNA Polymerase I in Saccharomyces cerevisiae
Source: PLoS One. 2013 Feb 20;8(2):e56793. doi: 10.1371/journal.pone.0056793 (PMC3577654; doi:10.1371/journal.pone.0056793)
Supplement: File S1 — Seven supplemental figures are provided. These data include additional controls for ChIP analyses, ChIP analysis of Snf5 association with the rDNA, analyses of rRNA synthesis rates in additional strains, controls for rDNA copy number and characterization of rRNA processing in WT and snf6Δ strains. Furthermore, thorough descriptions of methods employed for ChIP and rRNA synthesis measurements are included. (DOCX) [file pone.0056793.s001.docx]

**Figure S1.**

**
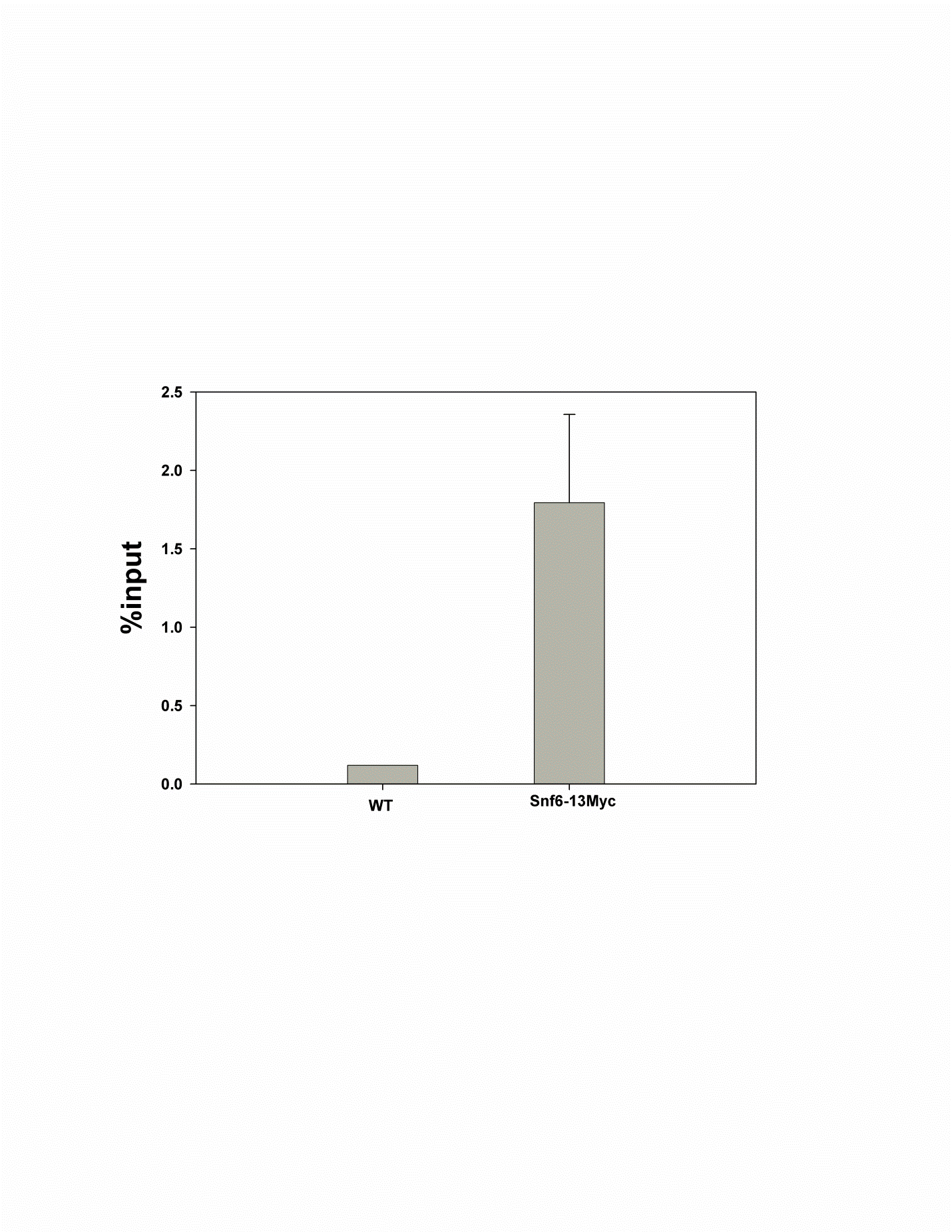
**

**Figure S1. Snf6-13Myc associates with the *GAL1* gene.** ChIP analysis of the coding region of *GAL1* showed clear association of Snf6p. This association is consistent with the literature. Furthermore, the magnitude of enrichment (~16-fold) relative to the untagged control strain is similar to that seen in the rDNA (Figure 2). Data were quantified from 2 10-fold dilutions per sample from duplicate cultures. Error = ± 1standard deviation.

**Figure S2.**


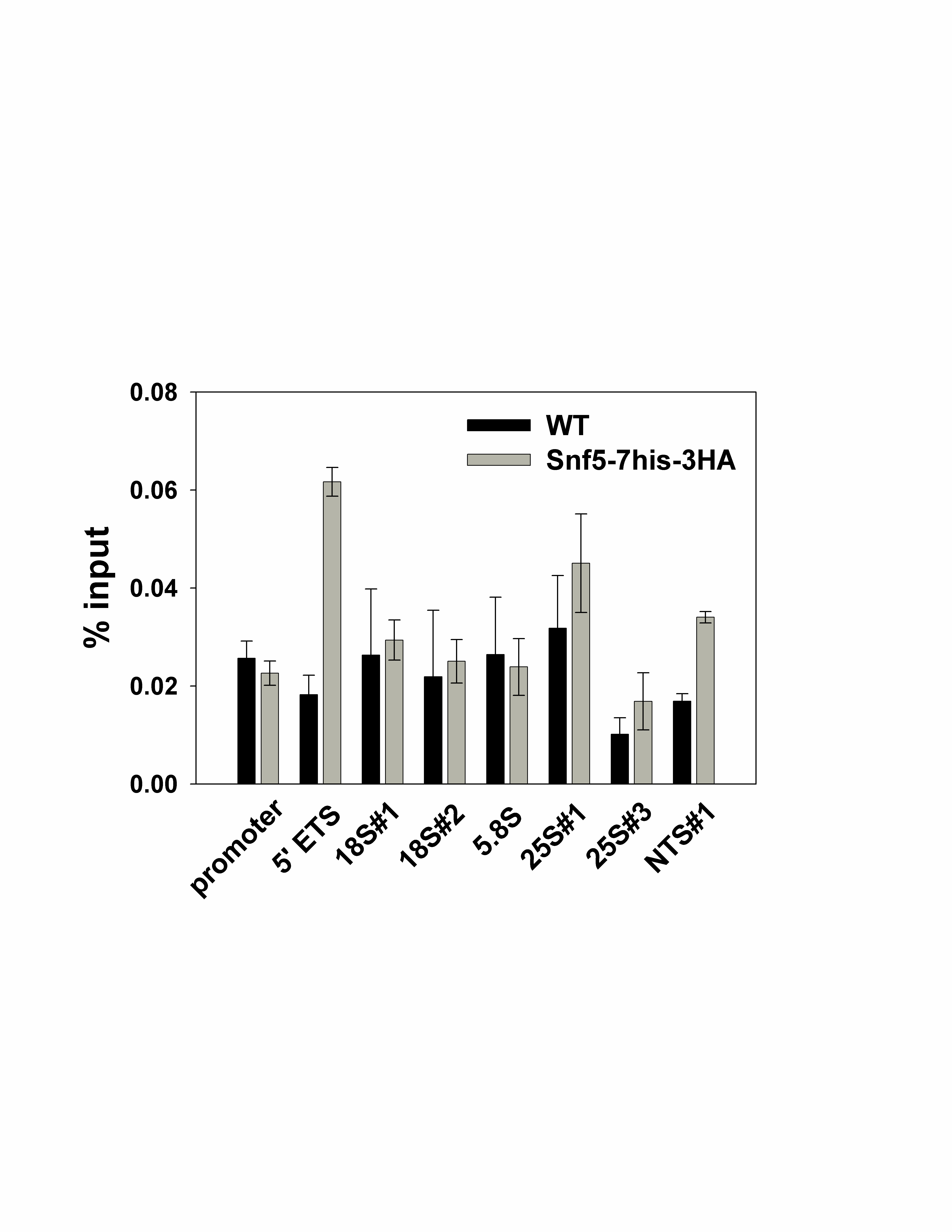


**Figure S2. Snf5-7his-3HA associates with the rDNA.** ChIP analysis indicates that Snf5p (DAS651) binds to the 5’ ETS of the rDNA with at least 2-fold higher signal than untagged control. Data were quantified from 2 10-fold dilutions per sample from duplicate cultures. Error = ± 1standard deviation.

**Figure S3.**


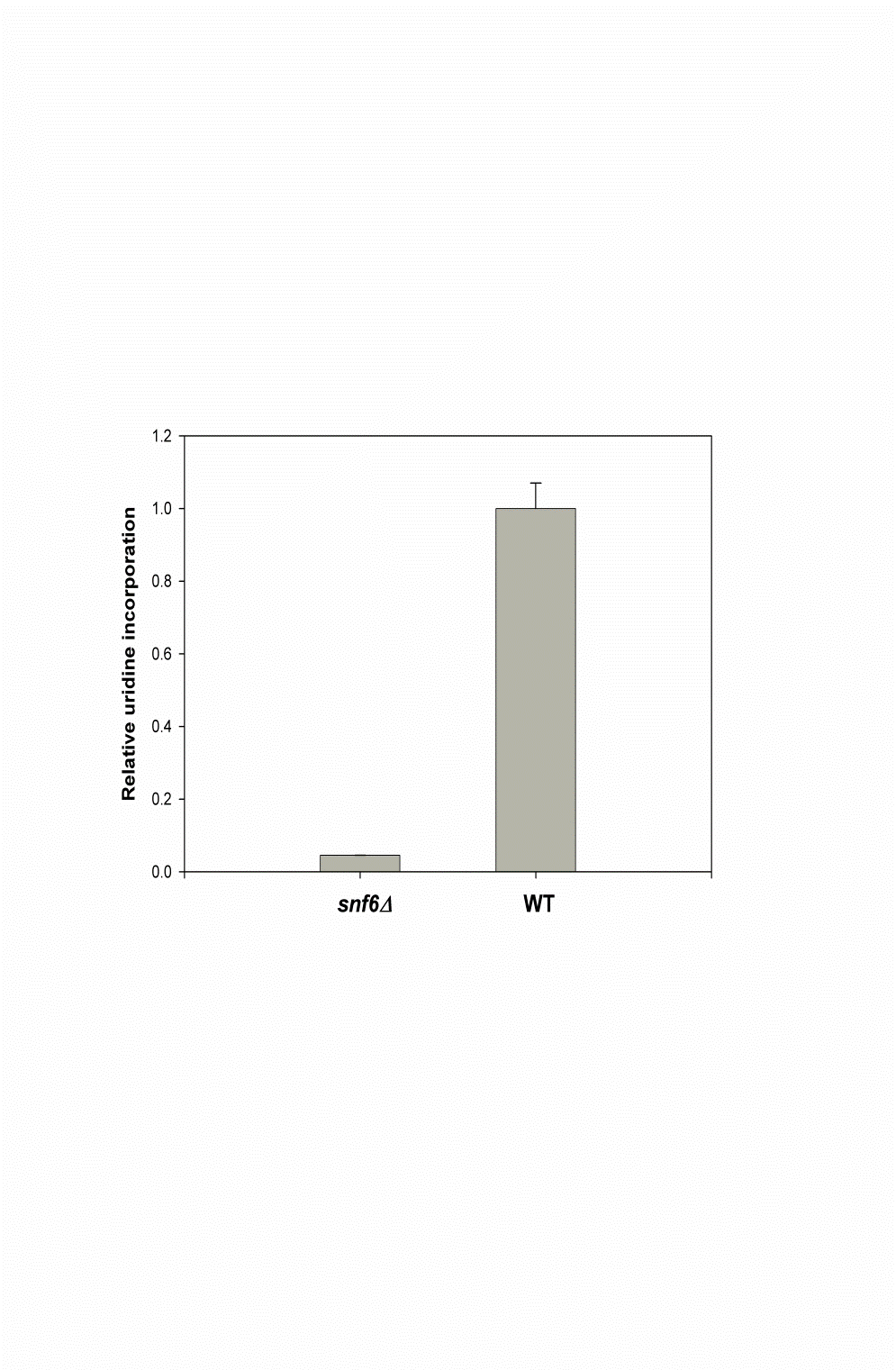


**Figure S3. Uridine incorporation shows that total RNA synthesis in *snf6∆* is significantly lower than in WT.** Relative RNA synthesis was measured by ^3^H-uridine incorporation in both WT and *snf6∆*. pRS316 plasmid was incorporated into both strains to obtain Ura^+^ phenotype, and the yeast cells were grown in SD-Ura media to exponential phase. One ml of yeast culture was incubated with 5µCi of ^3^H-uridine at 30°C for 5 min. Then 2.5 ml of 10% TCA containing 2.5mg/ml of cold uridine was added into the culture. After incubation on ice for >30 min, the mixture was filtered through a nitrocellulose membrane. The membrane was washed with 10ml cold TCA (5%), dried, suspended in scintillation cocktail, and quantified using a scintillation counter. Data were quantified from duplicate samples from duplicate cultures. Error = ± 1 standard deviation.

**Figure S4.**


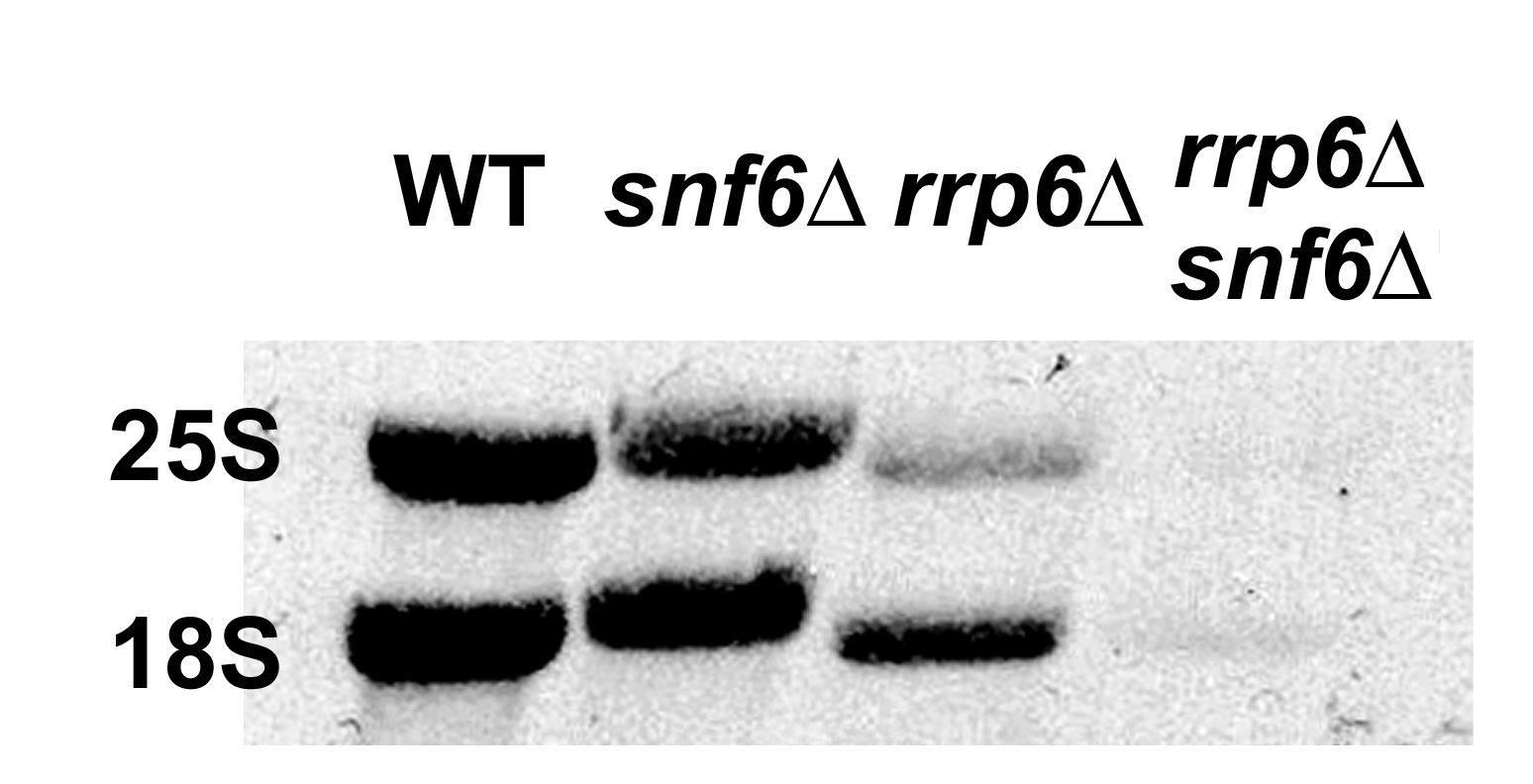


**Figure S4.** **Deletion of *RRP6* does not rescue rRNA synthesis in a *snf6Δ* strain.** Isotopic labeling was used to measure rRNA synthesis rates in WT, *snf6Δ*, *rrp6Δ* and *snf6Δ* *rrp6Δ* strains. Labeling with [methyl-^3^H]methionine was performed as described in the text for Figure 3. After transfer of labeled RNA to a blot, RNA was detected by autoradiography. This blot was exposed for 5 days, to visualize the faint signals from the RNA extracted from the double mutant cells. This overexposure resulted in non-linear detection of WT and *snf6Δ* RNA. The double mutant cells grow very poorly, consistent with impaired growth of both parental mutant strains. As expected for the poor growth rate, the rRNA synthesis rate in the *snf6Δ* *rrp6Δ* strain is much lower than in WT or parental mutant cells. These data confirm that mutation of the nuclear exosome does not rescue rRNA synthesis defects induced by mutation of *SNF6*.

**Figure S5**.

**
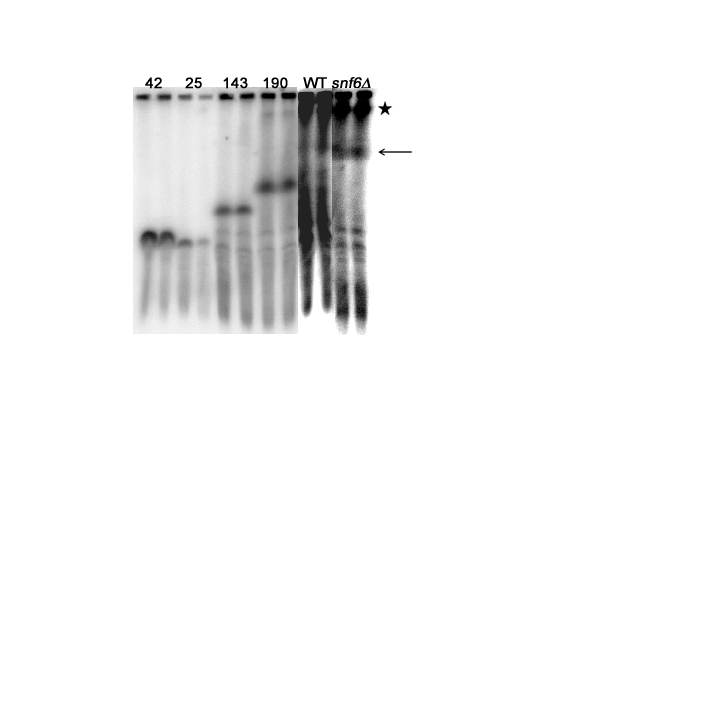
**

**Figure S5**. **rDNA copy number in *snf6∆* (DAS647) does not change compared to WT (DAS648).** CHEF followed by Southern hybridization was performed to show the position of chromosome XII where rDNA is located (indicated by an arrow for WT and *snf6∆*). Undigested cells are indicated by a star. Strains with known rDNA copy numbers [NOY1071, 25 copies; NOY886, 42 copies; NOY1051, 143 copies; and NOY1064, 190 copies [33] were included and designated with their rDNA copy numbers. Brightness and contrast were adjusted to visualize chromosome XII, but the position of the bands was not altered.

**Figure S6.**

**
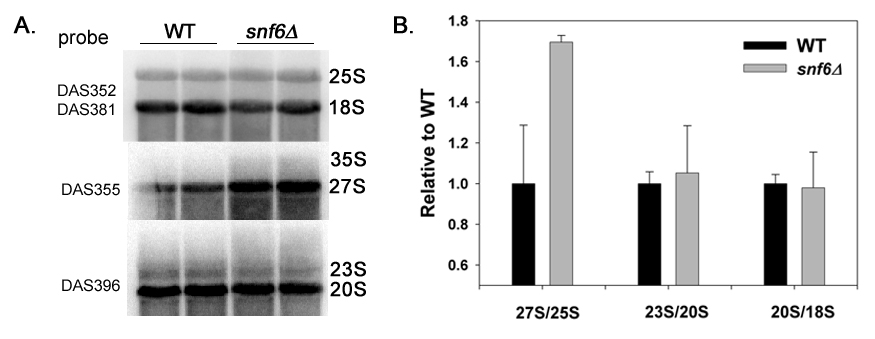
**

**Figure S6.** **rRNA processing is mildly impaired in *snf6Δ* cells compared to WT.** (A) RNA was isolated from two independent cultures of *snf6∆* and WT. 10 µg of RNA was run in a formaldehyde:agarose gel, transferred and probed with ^32^P labeled probes specific to precursor rRNAs and mature rRNA transcripts. (B) Precursor/product ratios (27S/25S, 23S/20S, and 20S/18S) in *snf6∆* normalized to WT. Data were quantified from duplicate cultures. Error = ± 1 standard deviation.

**Figure S7.**

**
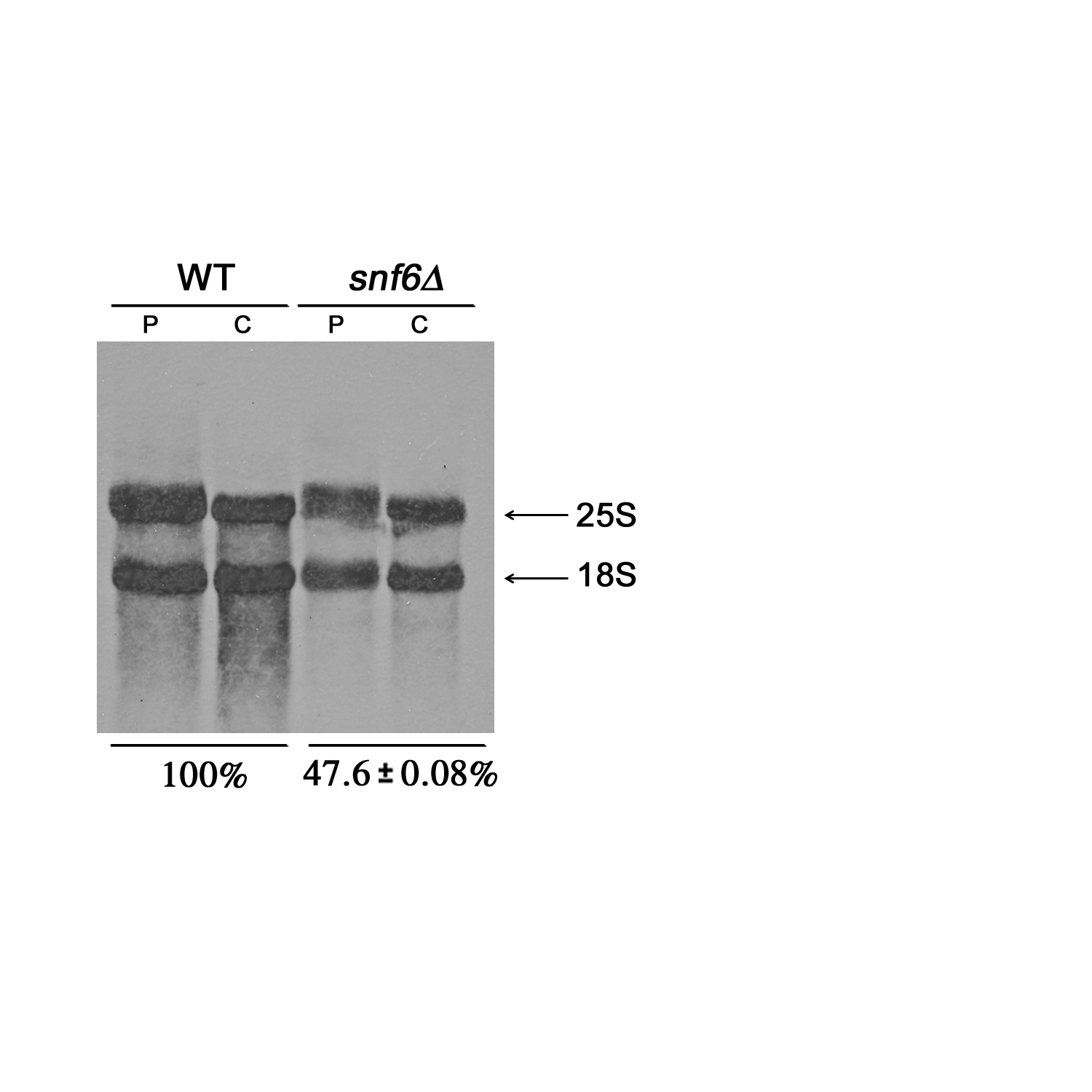
**

**Figure S7. Large defects in rRNA processing were not detected in *snf6Δ* relative to WT by ^3^H-methylmethionine pulse-chase**. RNA was purified from cells labeled with ^3^H-methylmethionine. The cells were either pulsed for 6min (labeled as ‘P’) or pulsed for 8min and then chased with cold methionine for another 5min (labeled as ‘C’). RNA was electrophoresed, transferred to a membrane and detected by autoradiography. 25S and 18S rRNA were cut from the membrane, and quantified with a scintillation counter. Relative expression levels are shown below the gel. Data were quantified from duplicate cultures. Error = ± 1standard deviation.

**Supplementary methods**

**Chromatin immunoprecipitation**

Yeast cells were grown in 50 ml of SD complete media at 30°C overnight until A_600_ reached ≈0.3-0.4. Growing cells were then treated with 1.5 ml of formaldehyde at room temperature for 6 min in the experiment of measuring Pol I occupancy of rDNA; however, 1 hr formaldehyde treatment was conducted to crosslink 7his-3HA-tagged Snf6p and Snf5p with rDNA. After crosslinking, 2.5 ml of Glycine (2.5M) was used to quench the formaldehyde for 10 min at room temperature. Cells were pelleted and washed in 1 X PBS twice and stored at -80°C until use.

his_7_-HA_3_ labeled proteins were immunoprecipitated by anti-HA 12CA5 antibody which was pre-treated with Prot-A sepharose (GE) for at least 3 hr at 4°C. The pre-treatment mixture contains 1ml FA lysis buffer (50 mM HEPES pH7.5, 140 mM NaCl, 1% TritonX-100, 0.1% Sodium deoxycholate, 1mM EDTA, 1mM PMSF added on the day of use, 1 tablet of protease inhibitor added on the day of use in 50 ml of FA lysis buffer), 22 µl of salmon sperm DNA, 50 µl of 10mg/ml BSA, 1.5µl of 12CA5 antibody per sample, and 20 µl of Prot-A sepharose per sample. It is unnecessary to perform the pre-treatment to immunoprecipitate Pol I complexes using polyclonal anti-A190. After 3hr pre-treatment, ProtA sepharose beads were centrifuged 2 min at 1200 x g and the beads were resuspended in double volume of FA lysis buffer.

Cell pellets were thawed on ice and suspended in 800 µl of FA lysis buffer. Cells were then broken using 400 µl of glass beads in a Fastprep (MP Biomedicals) (speed 4.5, 30 s, 4 times with 1 min rest between treatments). Cell lysates were then transferred to siliconized eppendorf tubes. DNA was sonicated using a Branson digital sonifier at 25% power, 0.9s pulse on, 0.1 s off, for 15 s. Samples were cooled on ice for at least 3 min and were then sonicated one more time. Finally, the samples were sonicated for another 30s with the same settings. Sonicated cell lysates were centrifuged for 10 min at 17,000 x g. Supernatant was harvested and cleared by centrifugation one more time.

Protein concentration was measured using a BCA kit (Pierce) following the manufacturer’s instructions. All samples were then diluted to the same concentration. Twenty µl of each sample were frozen immediately at -80°C as input. Two hundred µl of each sample were immunoprecipitated with 20 µl settled ProtA sepharose beads at 4 °C overnight.

After immunoprecipitation, sepharose beads were washed twice in 500 µl of FA-lysis for 10 min each. The same washes were then performed in order in high salt wash buffer (50 mM HEPE pH 7.5, 500 mM NaCl, 1% TritonX-100, 0.1% sodium deoxycholate, 1 mM EDTA) and deoxycholate wash buffer (10 mM Tris pH8.0, 250 mM LiCl, 0.5% NP-40, 0.5% sodium deoxycholate, 1 mM EDTA). Finally, the beads were suspended in 85 µl of 1 X TE with 1% SDS. The frozen input samples were suspended in 130 µl of 1 X TE with 1% SDS. Then samples were incubated at 65°C overnight to reverse the crosslinks.

The eluted DNA was purified by phenol-chloroform extraction. After overnight precipitation in ethanol, DNA pellet was suspended in 50 µl of water and run through PCR purification kit (Qiagen). Finally, DNA was eluted into 150 µl of water. Samples were frozen until use.

Both input and IP DNA were serially diluted in water. Three 10-fold dilutions were assayed for each sample. Each reaction contains 1 X buffer (Genechoice), 200 µM of each dNTP, 0.1mg/ml of BSA, 0.01 X SYBR green (Invitrogen), 0.025 U Taq (NEB), 4.5 µM of each primer, 9/20 (v/v) of DNA dilution in 20 µL of reaction buffer. ChIP signals were quantified by real-time PCR using an ABI 7900 HT system (Applied Biosystems). The sequences of primers and locations of amplicons are listed below. Data were quantified from at least two 10-fold dilutions per sample from duplicate cultures. The equation used to quantify percentage of IP relative to input at the same dilution is as follows: 100* 1/ (2^ ^(-∆Ct)^), ∆Ct = Ct^IP^- (Ct^input^ -3.32).

**Promoter RT1/2**

Forward 5’-ggcacctgtcactttggaa-3’

Reverse 5’-tcgccgagaaaaacttcaat-3’

**5’ETS RT3/4**

Forward 5’-aatagccggtcgcaagact-3’

Reverse 5’-tcacggaatggtacgtttga-3’

**18S#1 RT5/6**

Forward 5’-tggcctaccatggtttcaa-3’

Reverse 5’-cttggatgtggtagccgttt-3’

**18S#2 RT7/8**

Forward 5’-atcagcttgcgttgattacg-3’

Reverse 5’-aggcctcactaagccattca-3’

**5.8S RT33/34**

Forward 5’-aacggatctcttggttctcg-3’

Reverse 5’-cggaattctgcaattcacat-3’

**25S#1 RT9/10**

Forward 5’-aggatgctggcataatggtt-3’

Reverse 5’-cacccaaacactcgcataga-3’

**25S#3 RT15/16**

Forward 5’-cgaagcagaattcggtaagc-3’

Reverse 5’-cctgtctcacgacggtctaa-3’

**NTS1 RT17/18**

Forward 5’-gcaagatgaatagccagtgc-3’

Reverse 5’-tttacttcgccaaccattcc-3’

**5S RT27/28**

Forward 5’-agattgcagcacctgagtttc-3’

Reverse 5’-tggtaagagcctgaccgagta-3’

**NTS2 RT29/30**

Forward 5’-tagtccgcattgggatgttac-3’

Reverse 5’-gacggaaatacgcttcagaga-3’

**Predicted rRNA synthesis rate**

WT and *snf6∆* cells were cultured in SD-met medium overnight, and the A_600_ was measured over time. From the regression of A_600_ versus time, we calculated that the doubling time for WT was 1.3hr, and *snf6∆* was 2.6hr. Thus, the growth rate of WT was 1/1.3=0.77/hour, and *snf6∆* was 1/2.6=0.38/hour. The *snf6∆* grew 50% (0.38/0.77=50%) as fast as WT.

Total RNA was extracted from 10ml of WT and *snf6∆* cells (A_600_~0.3). RNA pellets were suspended in distilled water to a final volume proportional to the cell culture density to normalize for the number of cells harvested. Total RNA was then calculated from the A_260_ value of the resuspended RNA. We found that the total RNA content per cell in *snf6∆* is about 75% of WT.

Since most stable RNA is rRNA and the half-life of rRNA is very long, we can calculate the relative rRNA synthesis rates in two strains from these two values (growth rate and total RNA content). This calculation assumes minimal or no rRNA degradation. The predicted synthesis rate of rRNA for a mutant relative to WT level would be = [(RNA content relative to WT) * (growth rate relative to WT)]. Thus, Pol I transcription activity in *snf6∆* is estimated to be 75%*50%=37.5%, which is close to our quantification using isotopic labeling of the RNA in a pulse-chase experiment (Figure 3).
